# Control of metabolism by hypoxia and starvation and the consequences for the pattern of ecdysone secretion in *Manduca sexta*

**DOI:** 10.1242/jeb.251263

**Published:** 2026-02-06

**Authors:** Viviane Callier, Rebekah Pahren, Shu Wu, Victoria Bolling, H. Frederik Nijhout

**Affiliations:** Department of Biology, Duke University, Durham, NC 27708, USA

**Keywords:** Growth, Critical weight, Respiration, Endocrine cascade, Juvenile hormone, Lactate

## Abstract

When larvae of *Manduca sexta* pass a critical weight, they initiate an endocrine cascade that leads to molting and metamorphosis. The critical weight coincides with a leveling off of the metabolic rate, and we hypothesize that it is the size at which the metabolic needs of a growing body reach the maximum capacity of the tracheal system to deliver oxygen. We examined two simple predictions about the role of oxygen supply in the regulation of growth: first, that restricting access to oxygen by blocking spiracles should affect growth and metabolism, and second, that placing larvae in a hypoxic atmosphere shortly before they reached the critical weight should prematurely trigger cessation of growth and metamorphosis. When sets of spiracles were blocked, growth rate was reduced, as was the metabolic rate and the body size at metamorphosis. The effect of blocking the posterior-most spiracles was greater than that of blocking the anterior-most ones, suggesting the presence of a required abdominal factor. Contrary to expectations, placing larvae in hypoxia a day before they reached the critical weight delayed the molt significantly, suggesting that hypoxia is not a trigger for the initiation of metamorphosis. Nevertheless, an increase in lactate levels in the second half of the final instar, and the leveling off of metabolic rate, indicate that a metabolic shift occurs at the critical weight. Ecdysone secretion in starved and hypoxic larvae was delayed by about 2 days relative to feeding controls, which explains the delayed timing of the metamorphic molt.

## Teaser

**Summary:** Hypoxia in *Manduca sexta* does not induce a premature molt but reduces metabolic rate and delays the secretion of ecdysone. An increase in lactate is a better indicator of oxygen insufficiency than hypoxia inducible factor (HIF) expression.

## INTRODUCTION

The endocrine cascade that initiates metamorphosis in the tobacco hornworm, *Manduca sexta*, is triggered when the last instar larva reaches a critical weight (Davidowitz, D'Amico, and Nijhout, 2003; Nijhout and Williams, 1974a). This cascade begins with the decline in juvenile hormone (JH) (Nijhout and Williams, 1974a), whose presence inhibits the secretion of the molting hormone, ecdysone (Nijhout and Williams, 1974a; Rountree and Bollenbacher, 1986; Watson and Bollenbacher, 1988). Once JH is cleared, the ecdysone-regulatory system is enabled, and ecdysone secretion then occurs via a photoperiodic gated signal (Truman, 1972; Truman and Riddiford, 1974; Dominick and Truman, 1985). The critical weight was initially defined operationally as the mass after which no further feeding or nutrition is necessary for a normal time course of the endocrine cascade and metamorphosis. Under standard rearing conditions, this critical weight occurs at about the middle of the last larval instar, when the larva is about half the mass it will eventually grow to. Thus, larvae starved at the critical weight undergo normal metamorphosis at the same time as feeding controls, albeit at half the body mass, and develop into half-sized adult moths (Nijhout and Grunert, 2010; Grunert et al., 2015).

Physiologically, the critical weight is determined by the oxygen delivery capacity of the tracheal system. The tracheal system is lined with non-living cuticle, just like the exoskeleton. Its dimensions are set at the time of the molt when the old lining is replaced by a new larger one. In last-instar larvae of *M. sexta*, body mass increases about 10-fold, but the size of the tracheal system that supplies oxygen to the growing tissues is fixed. As the larva grows, oxygen consumption initially increases linearly with body mass, but then levels off when the larva passes the critical weight (Callier and Nijhout, 2011; Callier and Nijhout, 2013; Harrison et al., 2018). This suggests that the endocrine cascade that follows attainment of the critical weight may be triggered by oxygen insufficiency, when the increasing demand for oxygen of the growing tissues exceeds the capacity of the tracheal system to deliver more.

The endocrine cascade is thought to be controlled by the brain via the secretion of tropic hormones: the prothoracicotropic hormone (PTTH) that controls ecdysone secretion by the prothoracic glands, and allatotropins and allatostatins that control secretion of JH by the corpora allata (Bowers and Martinez-Pardo, 1977; Kataoka et al., 1989; Kramer et al., 1991; Agui et al., 1979; Smith and Rybczynski, 2012). Oxygen limitation must therefore somehow affect this neurosecretory activity of the brain. Here, we investigated two problems associated with this simple hypothesis. First, it is well known that larvae that are starved so they never reach the critical weight will, nevertheless, eventually secrete ecdysone and metamorphose, although with a significant delay (Davidowitz, D'Amico, and Nijhout, 2003; Nijhout and Williams, 1974b). Second, we show new data that when larvae are put in hypoxia before they reach the critical weight, this does not immediately trigger the endocrine cascade and begin metamorphosis as expected but, instead, they are much delayed and begin metamorphosis several days after normoxic controls.

## MATERIALS AND METHODS

Larvae of *Manduca sexta* (Linnaeus 1763) were reared on a standard diet (Table S1) at a constant temperature of 25°C under a long day (16 h light, 8 h dark) photoperiod. All experiments were performed in a constant-temperature environmental room held at 25±0.2°C. Hypoxic environments during growth were maintained by pressurized cylinders with a specific percentage mixture of oxygen and nitrogen (AirGas, Durham, NC, USA) and regulators that produced a flow rate of 100 ml ml min^−1^ through airtight flow-through chambers (Billups-Rothenberg, Inc., San Diego, CA, USA), monitored with a pair of floating ball flow meters (Gilmont GF2260). The oxygen concentration in the chambers was monitored continuously with a pair of oxygen meters (Gravity SEN0465 and SEN0496). Oxygen consumption was measured by placing larvae in a sealed 100 ml chamber that was flushed twice, 5 min apart, with the test gas mixture, and equilibrated for an additional 2 min before measurement began. Carbon dioxide was absorbed by 10 g of soda lime granules dispersed along the length of the cylinder. Oxygen uptake was measured by movement of a 0.2 ml drop of dyed water in a horizontally-held 2 ml pipette inserted in a gas-tight stopper at one end of the chamber. For each individual, measurements were made at 5 min intervals until 1.5 ml of gas had been absorbed. Identical control chambers without animals were placed next to the experimental chamber. In no case did we detect changes in the gas volume of the control chambers. Carbon dioxide production was measured by placing larvae in a flow-through cylinder of 75 ml volume. Air was pumped through at a rate of 100 ml min^−1^ (using a Sable Systems MFS-5 Mass Flow System) after passing through a 600 ml cylinder filled with soda lime pellets to remove all CO_2_. The gas was then passed through a Licor LI-820 CO_2_ analyzer, which reported the CO_2_ concentration in parts per million (ppm). Conversion from ml gas to moles was done as follows: taking the universal gas constant *R* as 0.0821 l atm K^−1^ mol^−1^, at 1 atm pressure (*P*) and 25°C (298K), *C*=*P*/*RT*=1/(0.0821×298)=0.04087 mmol l^−1^ and thus for 1 ml, the number of moles is 1/0.04087=24.47 mmol. For conversion of CO_2_, measured in ppm, the fraction volume of CO_2_ was calculated as ppm/10^−4^ ml min^−1^, so 1 ppm gives 2.447×10^−3^ mmol min^−1^. Ecdysone assays were done by ELISA (Fisher/Invitrogen), following the manufacturer's instructions. Lactate assays were done using the l-Lactate assay kit (MAK329, Sigma-Aldrich). Statistical analyses were done using JMP Pro 17 (SAS Institute, Cary, NC, USA).

## RESULTS

At the end of the larval feeding phase in *M. sexta*, a small PTTH-stimulated rise in ecdysone (rising to 25–35 ng ml^−1^ over a period of about 12 h; Bollenbacher et al., 1975; Wolfgang and Riddiford, 1986) causes the cessation of feeding and growth, and entry into the wandering stage. This is followed 3 days later by a much larger peak of ecdysone (rising to about 250 ng ml^−1^; Baker et al., 1987) that stimulates the molt to the pupal stage. Entry into the wandering stage is detected in two ways: first, larvae stop feeding and do a gut purge by which they lose most of their gut contents and decline in wet mass and initiate a day-long period of persistent locomotion; and second, the tissues along the dorsal midline become transparent and pink in coloration, so that the dorsal vessel (the heart) can be clearly seen. We take the time and mass at which a larva enters the wandering stage as the endpoint of its growth phase. The wandering stage lasts 5 days, after which the larva pupates.

### Limiting oxygen access by blocking spiracles

If the oxygen-delivery capacity of the tracheal system is a limiting factor in growth and size determination, then decreasing this capacity should result in earlier metamorphosis at a smaller body size. Air enters the tracheal system via nine pairs of spiracles, one pair on the first thoracic segment (T1) and eight pairs on abdominal segments 1 to 8 (A1–A8). To limit access of the tracheal system to atmospheric air, we blocked two pairs of spiracles with wax, either the anterior-most pair (T1+A1) or the posterior-most pair (A7+A8).

**Fig. 1.**
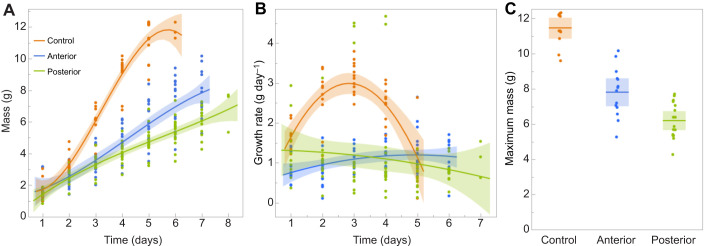
**Effect of spiracle blockage on growth and size of *Manduca sexta* larvae.** (A) Growth trajectories of larvae with two pairs of anterior or posterior spiracles blocked. (B) Growth rates of larvae with two pairs of anterior or posterior spiracles blocked. Lines are polynomial regressions, shaded regions are 95% confidence limits. (C) Peak sizes of larvae in A; bar is mean and shaded region shows 95% confidence limits. Significance of differences; *t*-test, one sided, unequal variances: control versus anterior *P*<0.001; anterior versus posterior *P*<0.001.

In a purely diffusion-limited system, one would expect that blocking 2/9=22% of spiracle area would result in a growth rate reduced to 78% of normal. Instead, growth rate was reduced to 41% (anterior blockage) and 30% (posterior blockage) of controls, indicating a disproportional effect on growth rate (Fig. 1). The duration of the growth phase was increased by 2 days, and the mean maximum size of the larva was reduced to 67% and 51% of controls for anterior and posterior blockage, respectively (Fig. 1). Thus, reduction in the intake area for air and oxygen has a disproportionate effect on both growth rate and the final size the larva will grow to.

**Fig. 2.**
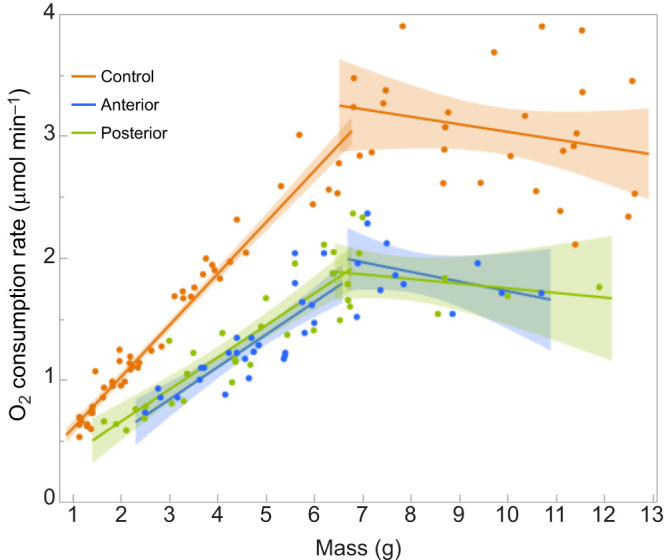
**Effect of spiracle blockage on metabolic rate.** Metabolic rate (measured as O_2_ consumption rate) trajectories are separately plotted before and after the critical weight of 6.5 g. Linear regressions with shaded regions showing 95% confidence limits.

The rate of oxygen consumption likewise decreased disproportionally after spiracles were blocked. Blocking two pairs of spiracles would reduce the area of oxygen intake to 78% and, if oxygen consumption was strictly diffusion limited and followed Fick's law, then one would expect a proportional decline. When spiracles were blocked, both the pre- and post-critical weight rates of oxygen consumption declined (Fig. 2). The post-critical weight oxygen consumption rate declined to 62% of control for both anterior and posterior spiracle blockages. The slope of the relationship between body mass and respiration rate for pre-critical weight larvae declined to 71% of control for anterior blockages and to 59% for posterior blockages.

Thus, a reduction in the number of active spiracles to 78% depressed metabolic rate to about 60% instead of the expected 78%. This decline approximately matches the decline in growth rate and final body size.

### Acute hypoxia

If the critical weight and the trigger to stop growing and metamorphose are determined by the oxygen delivery capacity of the tracheal system, then transferring larvae from a normoxic to a hypoxic environment when they are below the critical weight should prematurely induce the endocrine cascade that leads to the cessation of growth, molting and metamorphosis. We found the opposite to be true. The critical weight of the strain of *M. sexta* used in this study is approximately 6.5 g. When larvae weighing 5.0±0.3 g were transferred to 5% oxygen they entered the wandering stage after 5.9±1.4 days, compared with 3.3±0.5 days for normoxic controls, a relative delay of 2.6 days on average. If the onset of hypoxia were the signal that initiated the endocrine cascade, then we would expect a slightly earlier entry into the wandering stage rather than a delay.

**Fig. 3.**
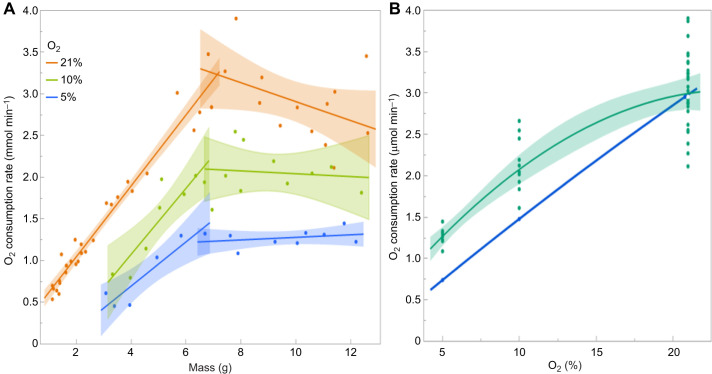
**Metabolic rate under hypoxia.** (A) Separate linear regressions of metabolic rate (measured as oxygen consumption rate) for larvae below and above the 6.5 g critical wieght under 21%, 10% or 5% O_2_, with shaded regions showing 95% confidence limits. (B) Post-critical weight oxygen consumption as a function of environmental oxygen. Green line, experimental data (polynomial regressions with shaded regions showing 95% confidence limits); blue line, ideal expectation if oxygen consumption were strictly proportional to environmental oxygen concentration.

We measured the rate of oxygen consumption of larvae 30–60 min after placement into a hypoxic environment with either 10% or 5% oxygen, and compared this with the pattern of oxygen consumption of control larvae at 21% oxygen (Fig. 3). The rate of oxygen consumption of control larvae increased linearly with body mass but stopped increasing in larvae larger than the critical weight, around 6.5 g. The rate at the critical weight was 0.12–0.13 ml min^−1^. The rate appears to decline after the critical weight but the 95% confidence envelopes in Fig. 3A show that the slopes of the regressions are not significantly different from zero. Thus, in control larvae, the oxygen supply becomes limiting at 0.12–0.13 ml min^−1^, and we assumed that a metabolic requirement above this value produced hypoxic stress that triggered the onset of metamorphosis (Callier and Nijhout, 2013; Callier and Nijhout, 2011). It also implies that in a hypoxic environment, this stress should manifest at a smaller body size.

Fig. 3A shows that when larvae were transferred to either 10% or 5% oxygen, their metabolic rate declined and their maximum metabolic rate declined with the oxygen concentration in their environment. The reduction in the rate of oxygen consumption was not strictly proportional to the level of atmospheric oxygen, however: at low oxygen levels the metabolic rate was somewhat higher than expected by strict proportionality (Fig. 3B). This suggests that in normoxia there may be excess capacity for metabolism that is not utilized. Perhaps the spiracles are not open 100% of the time under normoxia. At all three oxygen levels, the rate of oxygen consumption was constant in larvae larger than 6.5 g, which is the normoxic critical weight. This suggests that above this body mass, the tracheal system is conducting at its maximal velocity.

### Chronic hypoxia

**Fig. 4.**
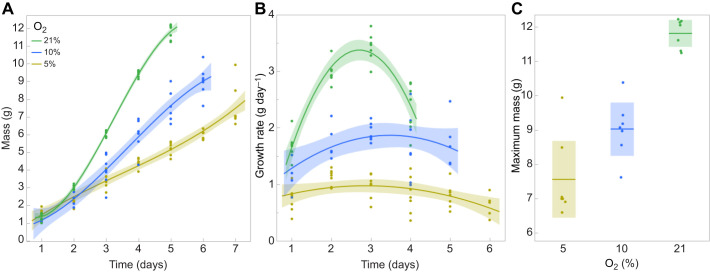
**Growth under chronic hypoxia.** Larvae were reared throughout the last larval instar under 21%, 10% or 5% O_2_. (A) Growth trajectories. (B) Growth rate as a function of body mass. Polynomial regressions with shaded regions showing 95% confidence limits. (C) Maximum mass based on data in A; bar is mean and shaded region shows 95% confidence limits. Significance of differences; *t*-test, one sided, unequal variances: 21% versus 10% *P*<0.001; 10% versus 5% *P*=0.012.

Acute hypoxia clearly depresses metabolic rate. We next investigated whether this was manifest in the larval growth rate under continuous hypoxia. We therefore studied the entire growth trajectories of last instar larvae that were continuously maintained in either 10% or 5% oxygen. Growth rates were indeed slower at lower oxygen tensions and the duration of the growth phase was increased (Fig. 4). In addition, at lower oxygen tensions, larvae stopped growing and metamorphosed at smaller body sizes.

The growth rate of control larvae during the last larval instar is a hump-shaped curve (Fig. 4B), with a peak rate at approximately the critical weight, corresponding to an inflection point in the growth curve (Grunert et al., 2015). In hypoxia, the mean growth rates were reduced and largely independent of body mass, with mean growth rates of 1.5 g day^−1^ in 10% oxygen and 0.99 g day^−1^ in 5% oxygen, compared with an average rate of 2.3 g day^−1^ in normoxia. The mass-specific growth rates declined with body mass, and likewise declined with declining oxygen concentration (Fig. 4B). The highest mass-specific growth rates at 2 g body mass were 1.0, 0.6 and 0.4 day^−1^ at 21%, 10% and 5% oxygen, respectively. Likewise, the maximum body size declined with decreasing oxygen concentration (Fig. 4C).

### Starvation delays entry into the wandering stage and lowers metabolic rate

**Fig. 5.**
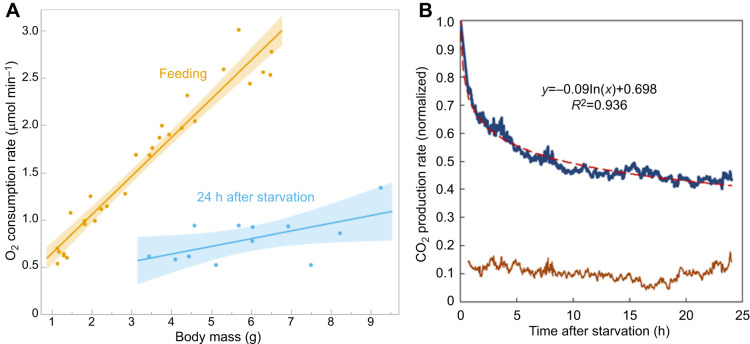
**Effect of starvation on metabolic rate.** (A) Starvation over a wide body mass range reduced O_2_ consumption to that normally found in a 2 g larva. Linear regressions with shaded regions showing 95% confidence limits. Slope of regression for starved larvae is not significantly different from zero. (B) Rate of CO_2_ production after starvation, illustrating a rapid decline with a good fit to a logarithmic decay function. Upper trace is the mean of 6 larvae of a range of body masses (4.5–11.6 g), normalized to the initial value of CO_2_ production. Lower trace is the standard deviation.

When we started to make measurements of metabolic rate, we found that larvae that had been taken away from their food had somewhat lower and more erratic rates of oxygen consumption than feeding larvae. We studied the effect of temporary starvation and found that after 24 h without food the metabolic rate had declined severely (Fig. 5A). Larvae starved for 24 h had the metabolic rate of a 2 g larva (roughly the mass at the beginning of the instar), independent of the mass at which they were starved. This starved metabolic rate was slightly lower than the maximal respiratory rate of larvae at 5% oxygen (cf. Figs 5A and 3A).

We measured the rate of decline of metabolic rate under starvation by continuously monitoring the production of CO_2_ (Fig. 5B). Metabolic rate declined rapidly to about 70% during the first hour, and then more gradually to approximately 40% of the initial value after about 20 h. The rapid decline during the first hour of starvation probably accounts for the apparently erratic metabolic rate of larvae when measured in the absence of food.

### Hypoxia and starvation delay ecdysone secretion

We showed above that larvae maintained at 5% oxygen eventually entered the wandering stage and metamorphosed normally, although they grew more slowly and metamorphosed at a much reduced body size. Most larvae growing at 5% oxygen never reached the critical weight; a few reached it less than 12 h before entering the wandering stage. It has been known for a long time that larvae that are starved below the critical weight will also enter the wandering stage after some delay relative to feeding controls (Callier and Nijhout, 2013; Nijhout and Williams, 1974b).

**Fig. 6.**
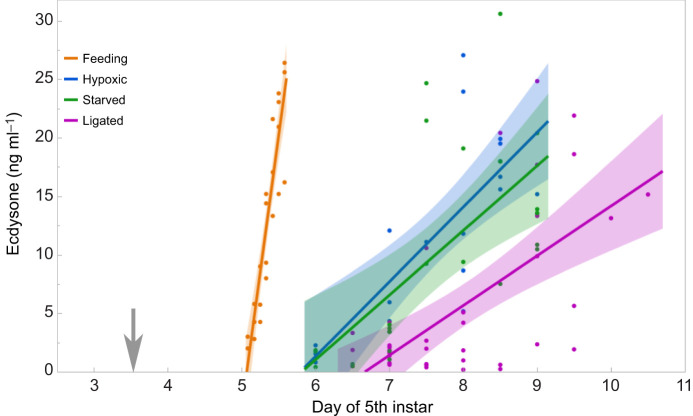
**Ecdysone titer in 5th instar larvae after treatment.** Larvae were placed in hypoxia, starved or neck-ligated (5.5−6.5 g) at the time indicated by the arrow. Control larvae were weighed and allowed to continue feeding. Lines are linear regressions with shaded bands showing 95% confidence limits.

Entry into the wanderings stage is triggered by a brief pulse of ecdysone that rises to 20–30 ng ml^−1^ over a period of about 12 h and then decays to background (Riddiford, 1978; Bollenbacher et al., 1975; Wolfgang and Riddiford, 1986). The fact that both starved and hypoxic larvae eventually entered the wandering stage implies that both types must have secreted ecdysone at some time. We therefore measured the progress of ecdysone levels in larvae that were starved at 5.5–6.6 g and larvae put in hypoxia at 5.5–6.5 g. Controls were feeding larvae selected at 5.5–6.5 g and allowed to continue feeding. The results are shown in Fig. 6, where we plot the rising phase of the ecdysone titers of control, starved and hypoxic larvae. Included in Fig. 6 are ecdysone titers for neck-ligated animals from a previous study (Callier and Nijhout, 2013).

Ecdysone began to rise in starved and hypoxic larvae about 2 days after feeding controls (Fig. 6), and rose more slowly than in control larvae. The starved and most of the hypoxic larvae never reached the critical weight, which is the normal trigger for brain-centered ecdysone secretion (Callier and Nijhout, 2013). In neck-ligated larvae, ecdysone began to rise about 3.5 days after feeding controls and rose more slowly than was seen in larvae that were hypoxia treated or starved. The rise in ecdysone levels in neck-ligated larvae indicates the timing of brain-independent ecdysone secretion. It is possible that ecdysone secretion in hypoxic and starved larvae was at least in part due to brain-independent secretory activity of the prothoracic glands as suggested by a previous study (Callier and Nijhout, 2013).

### Hemolymph lactate levels rise after the critical weight in normoxia

**Fig. 7.**
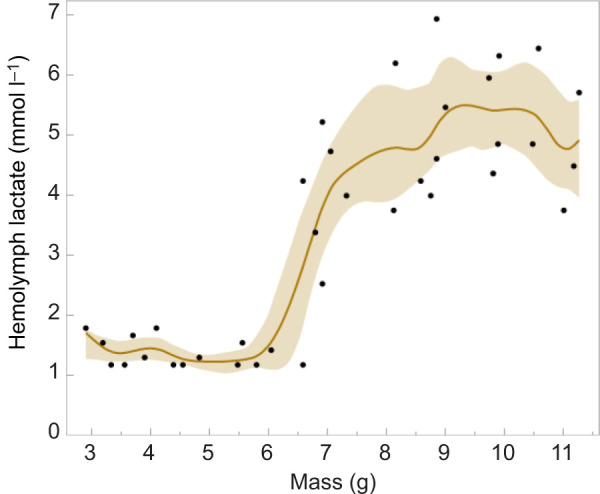
**Hemolymph lactate concentration before and after larvae pass the critical weight of 6.5 g.** Spline fit (λ=0.002) with shaded band showing 95% confidence limits. Significant difference of means before and after 6.5 g: *P*<0.001, *t*-test, unequal variances, one-sided.

Lundquist et al. (2018) suggested that lactate might be a more reliable indicator of hypoxic stress than hypoxia inducible factor (HIF) expression. Accordingly, we measured hemolymph lactate levels in 5th instar larvae of *M. sexta* throughout the instar. As larvae passed the critical weight lactate levels in the hemolymph rose from an average of 1.4 mmol l^−1^ to 4.8 mmol l^−1^ (Fig. 7). Together with the leveling off of metabolic rate, the increase in lactate after critical weight indicates that a metabolic transition occurs at the critical weight.

## DISCUSSION

Under ideal conditions, *M. sexta* larvae initiate the endocrine cascade that leads to metamorphosis when they pass the critical weight in the last larval instar. In the strain of *M. sexta* used in this study, the critical weight is about 6.5 g and is reached at about the middle of the third day of the 5-day-long last larval instar. On the 5th day, larvae secrete a brief pulse of ecdysone that triggers cessation of feeding and the beginning of the wandering stage. After larvae reach the critical weight, the endocrine cascade that leads to entry into the wandering stage proceeds without the need for further feeding or growth.

Physiologically, the attainment of the critical weight coincides with (a) a drop in JH, (b) independence of developmental timing from any further nutrition and (c) the leveling off of metabolic rate. In *M. sexta*, the rate of oxygen consumption increases proportionally with body mass until the critical weight is reached, at which point it levels off (Callier and Nijhout, 2011). After the critical weight, growth continues but the rate of oxygen consumption does not rise with the increase in body mass. We showed previously that larvae placed in hypoxia enter the wandering stage at a smaller body mass and metamorphose to a smaller adult than larvae reared in normoxia (Callier and Nijhout, 2011). We interpreted this result as indicating that at lower oxygen tensions the critical weight was proportionally lower so that the endocrine cascade that led to the cessation of growth and metamorphosis was triggered at a smaller body size.

The present study started as a simple attempt to test the hypothesis that oxygen limitation triggered the physiological cascade that leads to the cessation of growth and the metamorphic molt in *M. sexta*. The findings reported here that blocking of the spiracles, which reduces the area over which oxygen can enter the respiratory system, reduced the rate of growth and resulted in a premature cessation of growth and a smaller size at metamorphosis (Fig. 1) supported this hypothesis.

We expected that the reduction in growth rate and metabolic rate would be proportional to the fraction of spiracles blocked. However, we found that blockage of the area for oxygen intake had a lower than proportional effect on both growth rate and metabolic rate. We had also expected that blocking the anterior spiracles would have a greater effect on the reduction of growth rate and body size. This is because their proximity to the head and brain, the principal endocrine control centers, would increase the length of the diffusion path for oxygen, which would now start at the 2nd abdominal segment. Instead, we found that blocking the posterior-most spiracles had a greater effect (Fig. 1). This finding suggests that something from the abdomen may be required for normal growth and time course to metamorphosis. We previously showed that a brain-independent abdominal factor is required for normal growth of the wing imaginal disks in *M. sexta* prepupae (Tobler and Nijhout, 2010).

We had previously shown that larvae growing under reduced oxygen tension appear to have a lower critical weight and metamorphose at smaller body sizes (Callier and Nijhout, 2011). We therefore reasoned that placing a pre-critical weight larva in a reduced oxygen atmosphere should prematurely trigger the physiological processes that lead to the cessation of growth and metamorphosis. Instead, we found that both events were delayed. The reason for this appears to be that the metabolic rate of larvae in a reduced oxygen atmosphere was reduced, in proportion to the oxygen concentration (Fig. 3). Although this is in accord with the expectation that the metabolic rate is limited by the ability of the tracheal system to supply oxygen, the fact that oxygen limitation did not trigger the cessation of growth suggests that this is not a sufficient or perhaps even a necessary signal for triggering the ecdysone pulse that terminates growth.

In many animals, hypoxic stress is sensed by HIF, which is stabilized in low oxygen conditions. In hypoxia-reared *Drosophila*, knocking down HIF in the prothoracic glands (the source of ecdysone) causes the animals to grow much larger than they would otherwise, suggesting that hypoxia sensing in those glands is necessary for triggering molt at a smaller size (Kapali et al., 2022).

However, hypoxic rearing does not appear to mimic whatever occurs at the critical weight in normoxic conditions: larvae placed in acute hypoxia delay, rather than accelerate, the transition to metamorphosis. And in *M. sexta*, HIF-α protein in the tracheal tissue is decreased, rather than increased, in late 5th instar larvae relative to early 5th instars (Lundquist et al., 2018), suggesting that the late 5th instar larvae are not limited by their ability to take in enough oxygen through the trachea.

Nevertheless, in *M. sexta*, lactate (Fig. 7) increases after the critical weight and oxygen consumption levels off. Similarly, in *Drosophila*, lactate dehydrogenase is highly active during larval development and peaks during the third (final) instar before declining during metamorphosis (Rai et al., 2024). A recent preprint by Zhao et al. (2024 preprint) shows that in the final instar of *Drosophila*, the wing imaginal discs shift from reliance on oxidative phosphorylation to glycolysis. In other words, a metabolic shift occurs in the final instar in both *Manduca* and *Drosophila*; it just does not seem to be caused by a constraint on the ability to take in enough oxygen.

Zhao et al. (2024 preprint) show that as the *Drosophila* larvae progressed through the final instar, the imaginal discs had progressively more HIF-1α activity, suggesting that the discs were becoming hypoxic as a result of growth-induced demand. They found that HIF-1α acts to inhibit TOR signaling in the imaginal discs, slowing down growth and thus preventing oxygen demand from exceeding supply. Their findings suggest that mild hypoxia occurs not globally in the body, but in each individual growing imaginal disc. The deceleration of growth of the imaginal discs in response to mild local hypoxia was also regulated locally by TOR. When the researchers drove TOR activity, the size of the discs increased but there was evidence of cellular stress and wing crumpling, probably due to a mismatch between oxygen demand and supply.

This local regulation of growth by oxygen sensing in the imaginal discs is fascinating. The findings raise a few questions. The first is how this local (tissue-level) control of growth interfaces with systemic hormonal signals that coordinate the growth and transition of all imaginal discs together at metamorphosis. Indeed, while some growth of body parts occurs autonomously, there is also evidence that damaged wing discs emit signals to allow catch up growth and coordinate the growth of tissues to ensure a correctly proportioned adult (Parker and Shingleton, 2011; Stieper et al., 2008). The mechanisms by which growth is coordinated among developing tissues remain largely unknown. It is the interaction between these local tissue-level mechanisms (growth-induced hypoxia) and the systemic mechanisms that coordinate tissue growth with developmental transitions that still need to be elucidated.

The findings of Zhao et al. (2024 preprint) also raise questions about hypoxic rearing. If local tissue hypoxia (and resulting inhibition of TOR) is the main factor regulating the cessation of imaginal disc growth, then rearing larvae in hypoxic conditions should lead them to reach the threshold at which HIF-1α acts to inhibit TOR at a smaller size. But the slowing or cessation of growth does not seem to be sufficient, on its own, to trigger metamorphosis: hypoxia-reared larvae, as well as larvae introduced into acute hypoxia, both show variable delays in initiating metamorphosis (e.g. our findings presented here and in Callier et al., 2013).

The slower growth and maturation delay observed in hypoxia-reared *Drosophila* appears to be caused, independently from nutrient deprivation or from HIF-1α, by a decrease in the expression of epidermal growth factor (EGF) in the prothoracic glands (Turingan et al., 2024). Thus, we are beginning to understand the mechanisms by which hypoxic rearing affects ecdysone secretion from the prothoracic gland and results in a smaller final body size. These hypoxic response pathways appear distinct from the mechanisms involved in the response to poor nutrition or higher temperature, two other causes of small body size.

In normoxia, the findings of Zhao et al. (2024 preprint) suggest that local hypoxia in each imaginal disc regulates the cessation of growth in that disc via HIF-1α and TOR. Campbell et al. (2024) showed that in normoxia, knocking down HIF-1α in the prothoracic gland only marginally delays the initiation of metamorphosis and results in increased body size in females but not in males. Because the effect of knocking down HIF-1α in the prothoracic gland was very clear in hypoxia but only subtle in normoxia, it was concluded that HIF-1α activity in the prothoracic gland is involved in metamorphosis initiation only in hypoxia but not in normoxia (Campbell et al., 2024).

However, it is possible that low levels of HIF-1α in the knock-down experiment (Campbell et al., 2024) are sufficient for physiological function in normoxia: Heidarian et al. (2025) show that the canonical sima/HIF-1α mutant fly harbors a hypomorphic allele and is able to complete larval and pupal development, but a new sima/HIF-1α mutant carrying a CRISPR-induced frameshift mutation, which completely eliminates sima/HIF-1α activity, dies during the second larval instar. This suggests that even low levels of sima/HIF-1α can have physiological activity. Indeed, as HIF-1α works by being stabilized and accumulating under local tissue hypoxia, low levels of HIF-1α (in a HIF-1α knock-down) would potentially take longer to accumulate but eventually reach levels relevant for physiological activity. Thus, we cannot eliminate the possibility that HIF-1α activity in both the imaginal discs and the prothoracic gland plays a role in regulating the cessation of growth and transition to pupal stage in normoxia. The question that remains is, how is the cessation of growth in the wing discs connected to the systemic signals that regulate the metamorphic transition?

We see two broad possibilities: ‘bottom up’ or ‘top down’, which must somehow interact. The ‘bottom up’ possibility is that the growth-induced shift in metabolism in each imaginal disc sends a signal to the prothoracic gland to initiate the hormonal cascade. Mitochondria in the developing wing discs would be at the center of sensing growth-induced hypoxia and ideally situated to signal to the prothoracic gland that it is time to initiate the developmental transition to metamorphosis. In the last few years, evidence has accumulated showing that mitochondria are not just metabolic organelles but also signaling organelles that communicate across tissues to regulate cell fate, immunity and aging (Shen et al., 2024; Shen et al., 2022; Zhang et al., 2023). Thus one natural hypothesis to investigate is whether mitochondrial signals across larval tissues are playing a role in the coordination of growth and the metamorphic transition.

The ‘top down’ possibility is that the prothoracic gland tunes the growth and metabolism of imaginal discs via ecdysone secretion. Several studies have shown that ecdysone modulates the growth of wing discs (Delanoue, Slaidina, and Léopold, 2010; Herboso et al., 2015). We previously showed that baseline ecdysone levels are higher in hypoxia-reared *Drosophila* larvae (Callier et al., 2013) and that inhibiting ecdysone synthesis reduces the slowing effect of hypoxia on growth (Kapali et al., 2022). In *Drosophila*, ecdysone also regulates lactate dehydrogenase (Abu-Shumays and Fristrom, 1997), suggesting a possible mechanism to link the hormonal control of metamorphosis with the metabolic shift in the final instar. Muscles in the abdominal wall of caterpillars can be rich in lactate dehydrogenase (Greenlee and Harrison, 2005; Gade, 1975; Finn and Lockshin, 1981) and are thus reasonable candidates for the source of lactate. Lactate is increasingly recognized as an important signaling molecule in cell physiology (Certo et al., 2022; Pellerin et al., 2022), and as an epigenetic substrate (An et al., 2023) that can regulate gene expression. Its potential role in the initiation of metamorphosis remains an open question.

Finally, in working to understand how local tissue growth is coordinated with the systemic signals for developmental transitions, it is important to note that systemic signals are not exclusively produced by the prothoracic gland. In fact, the spiracle blockage experiments (Fig. 1) suggest that something in the abdomen is sensitive to the oxygen supply and is required for the timely secretion of ecdysone; its absence can significantly reduce growth rate and body size. Although the prothoracic gland and brain are the principal endocrine centers, this abdominal factor must also be considered when we try to understand how local, tissue-autonomous growth interfaces with systemic regulation of metamorphosis. The nature of this abdominal factor is currently under investigation.

## Supplementary Material

10.1242/jexbio.251263_sup1 Supplementary information
