# Supplementary material for: Control of metabolism by hypoxia and starvation and the consequences for the pattern of ecdysone secretion in Manduca sexta
Source: J Exp Biol. 2026 Feb 6;229(3):jeb251263. doi: 10.1242/jeb.251263 (PMC12912263; doi:10.1242/jeb.251263)
Supplement: Supplementary information [file jexbio-229-251263-s1.pdf]

Table S1. Artificial diet for *Manduca sexta*

ingredient	amount per liter		source **
Agar	12	grams	M
Wheat germ, Organic Lekithos *	120	grams	A
Casein	53.5	grams	B F
Torula year	23.8	grams	B F
Wesson salts	18	grams	B F
Cholesterol	5.25	grams	B
Sucrose	47	grams	local
Sorbic acid	3	grams	B
Methylparahydoxybenzoate	1.5	grams	S
Ascorbic acid	7.5	grams	S
Streptomycin	0.3	grams	S
Kanamycin	0.06	grams	S
Vanderzandt vitamin mix	0.75	grams	B
Propionic acid	0.75	ml	S
Linseed oil (raw)	7	ml	local
Formaldehyde (37%)	4.75	ml	S

* We used Lekithos brand organic wheat germ sourced from Europe. Most, and perhaps all, the wheat germ in the US is contaminated with methoprene, a juvenile hormone analog used as a growth regulator and disruptor for insect pest control. Juvenile hormone greatly affects the growth and the final body size of *Manduca*, and using animals feeding on diet with contaminated wheat germ that contains an insect growth regulator would invalidate nearly all the data reported in this paper.

**sources
A, Austrade, Inc.
B, Bio-Serv
F, Frontier Agricultural Sciences
S, Sigma
M, Moorhead & Co.

Journal of Experimental Biology • Supplementary information
